# Oral hygiene and periodontal health status among khat chewers. A case-control study

**DOI:** 10.4317/jced.53520

**Published:** 2017-05-01

**Authors:** Sadeq-Ali AL-Maweri, Mohammed AlAkhali

**Affiliations:** 1 Assistant professor, department of Oral Medicine and Diagnostic Sciences, Alfarabi colleges, Riyadh, Saudi; department of Oral Medicine, Faculty of Dentistry, Sana’a University, Yemen; 2 Associate professor, Department of periodontology, Faculty of Dentistry, Jazan University, KSA; Associate professor, Department of periodontology, Faculty of Dentistry, Sana’a University, Yemen

## Abstract

**Background:**

Habitual khat chewing is a widespread male habit in Southern Arabia and East Africa. The aim of this study was to assess the effect of Khat chewing on oral hygiene and periodontal health status among Yemeni male khat chewers.

**Material and Methods:**

This case- control study included 310 khat chewers, aged 20-65 years, and 72 age- and gender- matched non-chewers as controls. Periodontal examination included recording of dental plaque index (PI), gingival index (GI), probing pocket depth (PPD), attachment level (AL) and gingival recession (GR). Demographic data, oral hygiene practices, and duration, frequency and site of khat chewing were obtained using questionnaires. SPSS was used for data analysis. ANOVA and t- tests were used to compare groups.

**Results:**

Khat chewers had significantly higher mean PI, AL, GR scores than non-chewers (*P* < 0.05). However, no significant differences in the GI and PPD mean scores were observed between both groups (*p* > 0.05). Interestingly, the results showed that the chewing side had less PI, GI and PPD than non-chewing side. However, gingival recession was significantly higher in the chewing side. The duration of chewing showed a significant effect on PI, PPD, AL, and GR but had no effect on the gingival condition. Likewise, the frequency of chewing showed a significant effect on PPD, AL, and GR, but had no effect on other indices (PI and GI).

**Conclusions:**

The results of this study indicate that khat chewing have a detrimental effect on the periodontium.

** Key words:**Khat chewing, periodontal health, oral hygiene.

## Introduction

Habitual khat chewing is a widespread habit in East Africa and the south Arabian Peninsula. Khat, also known as Qat, Kat, and Mirra (in Kenya), is an evergreen plant (Catha edulis) that belongs to the Celastraceae family (1). People habitually chew its fresh leaves and twigs for their stimulating amphetamine-like effects (2,3). This habit is particularly highly prevalent in Yemen, Kenya, Ethiopia, Eretria, Somalia and Saudi Arabia (1,4). Khat use has recently spread with immigrants to Europe, North America, and Australia, becoming an international phenomenon (1,5). It is estimated that more than 20 million worldwide practice this habit regularly (4). People usually chew khat in special social gathering, locally known as khat session, which starts in the afternoon and lasts for several hours a day (2,5). Typically, it involves inserting and chewing around 100-200 gm of fresh leaves, forming bolus that is held in the buccal sulcus against the cheek on one side of the mouth while swallowing its juice (5,6).

Long term Khat chewing has been reported to have several detrimental health effects, such as cardiovascular diseases, gastrointestinal tract disorders, liver and renal toxicity as well as psychological and mental disorders (3). Additionally, khat use has been reported to have detrimental effects on dental and oral tissues that includes keratotic white lesions (7,8), mucosal pigmentations (8), plasma cell stomatitis (9) teeth attrition and discoloration (8), gingival recession (5,8,10), and Xerostomia (8,11). The association between khat chewing and periodontal diseases has long been debated. While most studies found a positive association between khat chewing and the occurrence of periodontal diseases (5,8,10,12-14), some did not report this association (15,16). Rosenzweig and Smith (17) were the first investigators to suggest a possible effect of khat chewing on periodontal tissues. In their study, the authors found that Yemeni Jews had higher rates of periodontal diseases compared to other ethnic groups. They attributed this to Khat use as many of these patients had chewed Khat before immigration to Israel. Further, in another large scaled study on Yemeni Khat chewers (n= 1001), it was found that the periodontal treatment needs, the attachment loss and the calculus index were significantly higher in the khat chewers (12). More recent studies have also reported khat chewing as a risk factor for periodontal diseases (5,10,13). On the contrary, Jorgensen and Kaimenyi (16) in their study on the association between periodontitis and khat did not find significant differences in the periodontal condition of Khat chewers and non-chewers. Likewise, in an *in-vivo* 20-day experimental gingivitis study, the authors observed that khat chewers had lower mean scores of plaque index, gingival index and bleeding on probing than non-chewers, suggesting a beneficial effect of khat on periodontal tissues (15).

In light of the above conflicting results, the present study was conducted to investigate the following: 1) the effect of khat on periodontal health and oral hygiene status among khat chewers compared to non-chewer control subjects 2) the effect of khat on the chewing side compared with the opposite non-chewing side 3) the association between the duration and frequency of Khat chewing and the periodontal health status.

## Material and Methods

This case- control study, conducted over 4 months period from May to September 2013, included a sample of 310 male khat chewers (aged 20-65 years), and 72 age- and gender- matched non-chewer control subjects. All subjects were randomly recruited from the out-patient dental clinics at Sana’a University, Yemen. Sample size calculation was done according to the expected prevalence of periodontitis and plaque accumulation among adult khat chewers (12), with a 95% confidence level and 0.05 acceptable level of error. Accordingly, the minimum sample size for this study was 210, which was rounded to 310 to decrease type I error and improve power of the study. The final recruited sample was 310 khat chewers and 72 non-chewer control subjects. The 1:4 ratio of controls (non-chewers) to cases (Khat chewers) was based on the estimated 80% prevalence of khat chewers in Yemen and the difficulty of finding non-chewer male subjects (5). The study was approved by the Research and Ethics Committee, Faculty of Medicine and Health Sciences, Sana’a University, Yemen, and written consents were obtained from the participants.

A history of Khat chewing for at least 5 years with a frequency of at least once a month and a minimum chewing session duration of 3 hours was used to define a Khat chewer (6). Non-chewers were employed as control subjects. A non-chewer was defined as a person who had never chewed khat or those who chewed no more than 5 times in their lifetimes. Exclusion criteria in both groups were: previous periodontal treatment in the last 3 months ; being under the age of 20 years; presence of any systemic diseases known to affect the periodontium; antibiotics intake in the last 3 months.

Khat chewers were divided into various subgroups according to: 1) the side of chewing: :ight chewers; left chewers; both side chewers. 2) the duration of chewing: chewing for more than 20 years; Chewing for 10-20 years; Chewing for less than 10 years. 3) the frequency of chewing: daily chewers; weekly chewers; rarely chewers. Daily khat chewers were defined as individuals who at the time of examination chewed khat daily. Weekly khat chewers were defined as individuals who at the time of examination chewed khat for once or more per week. Rarely chewers are those who chew khat but not in regular basis. Prior to clinical examination, demographic data such as the name and age, oral hygiene practices, and duration, frequency and site of khat chewing were obtained by using structured questionnaires.

-Clinical examination.

Clinical examination was performed by a single examiner using dental mirrors No.4, periodontal probes type Williams (0.5-mm tip), kidney dishes, masks and gloves, cotton (for dryness), and sonic scaler.

Oral hygiene and periodontal health status were assessed by the following parameters, recorded on all teeth:

1) Plaque index according to Silness and loe (18)

2) Gingival index according to loe and Silness (19)

3) Probing pocket depth (PPD): The distance from the gingival margin to the base of periodontal pocket depth using Williams periodontal probe.

4) Attachment level (AL) by using Williams’s periodontal probe and was carried out as follows:

a) Measuring the distance in millimeters from the free gingival margin (FGM) to the cementoenamel junction (CEJ). b) Measuring the distance from FGM to the bottom of the pocket at each site. c) AL was obtained by subtracting the first measurement from the second one (20).

For accurate measurement of AL, scaling was done for all patients with detected supragingival calculus (after measurement of PI, GI. PPD) to identify cementoenamel junction (CEJ).

5) Gingival Recession (GR): The distance from the CEJ to the free gingival margin was assessed in millimeters using Williams probe and was rounded to the lowest whole millimeter. Gingival recession was defined as the CEJ / free gingival margin distance when the gingival margin was located on the root. The measurements were recorded for all teeth except third molars.

Additionally, in order to compare the periodontal health status in chewing side vs non-chewing side, PLI, GI, PPD and GR were recorded in the posterior teeth (premolars and molars); anterior teeth and third molars were excluded.

Prior to the commencement of the study, a pilot study was carried out on 20 subjects for the purpose of examiner’s calibration. The correlation coefficient (r) of intra-examiner calibration for gingival index, probing pocket depth, attachment level and gingival recession were 0.86, 0.91, 0.93, 0.91, respectively. Training was also done on extracted teeth for detection of CEJ by using the same periodontal probe that was used in the study.

-Statistical Analysis: SPSS version 12.0 was used for data entry and analysis. Qualitative data were presented as frequencies and percentages, while quantitative data were presented as means and standard deviations. Students t test, analysis of variance (ANOVA) were used for comparison between groups, with *p*-values < 0.05 were considered statistically significant.

## Results

A total of 382 subjects participated in the study, 310 of whom were khat chewers and 72 were non-khat chewers. Of khat chewers, 67% were daily chewers and 45% reported practising the habit for 10-20 years. Most of subjects in both groups showed poor oral hygien practices, with only 6% of khat chewers and 38% of controls rported brushing their teeth regularly ( Table 1, Table 2) .

**Table 1:**
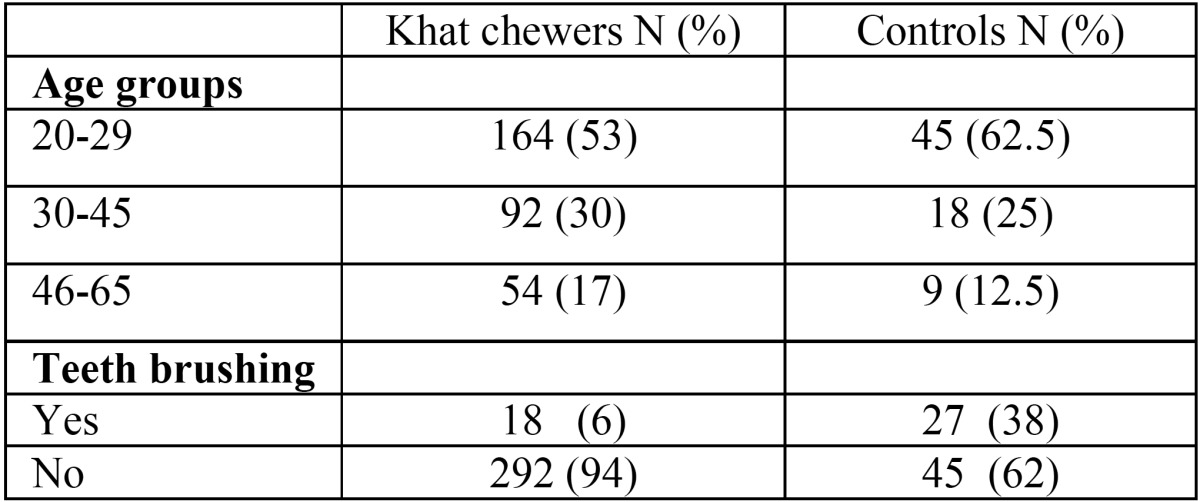
Demographic data of the study groups.

**Table 2:**
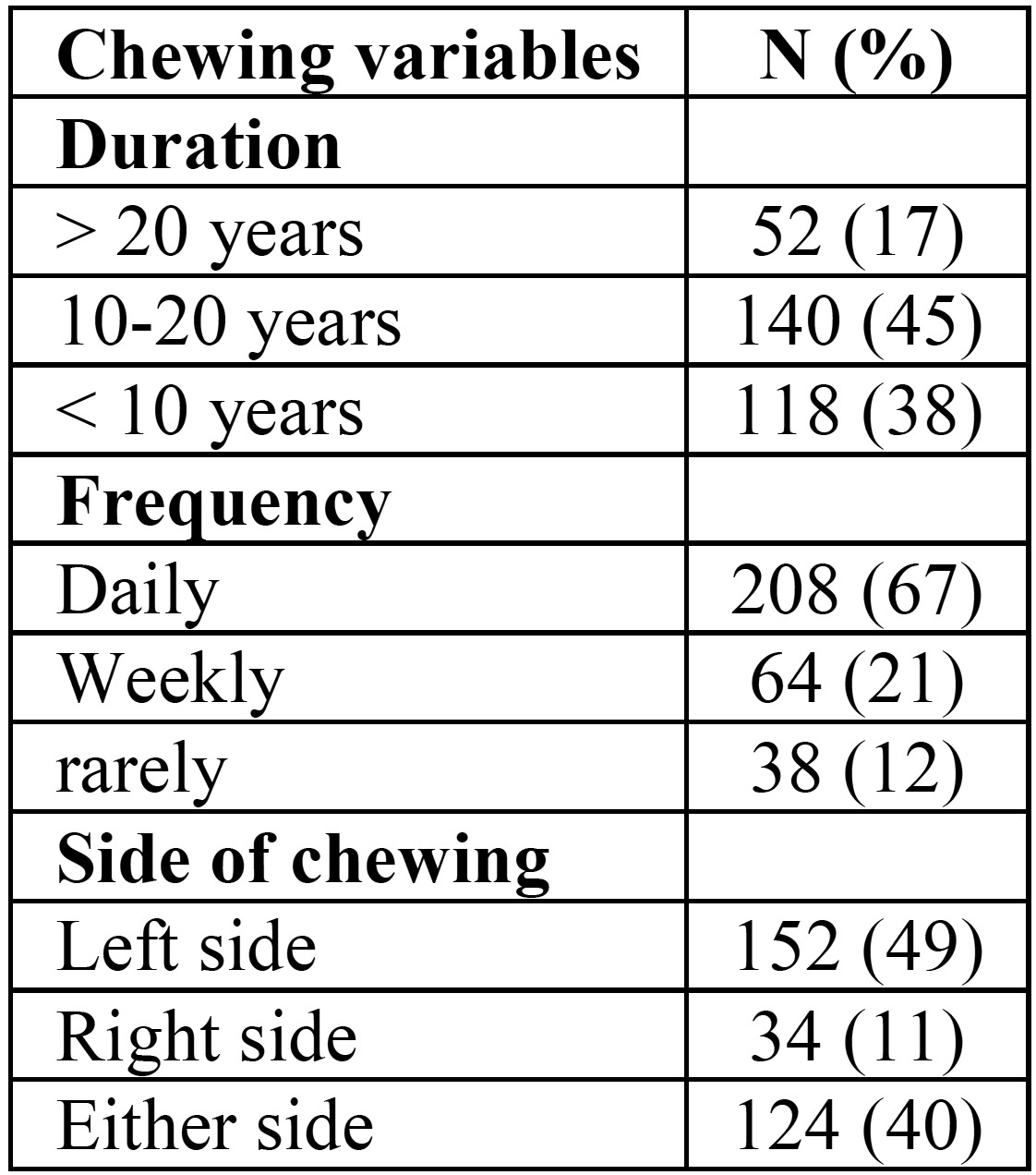
Chewing variables among Khat chewers group.

 Table 3 illustrates the mean scores of periodontal parameters in both groups. The mean PI, AL, GR scores were significantly higher among Khat chewers (1.01, 0.87, 0.38, respectively) than non-chewers (0.80, 0.36, 0.19, respectively ). However, no significant differences in the GI and PPD mean scores were observed between the groups (*P* > 0.05).

**Table 3:**
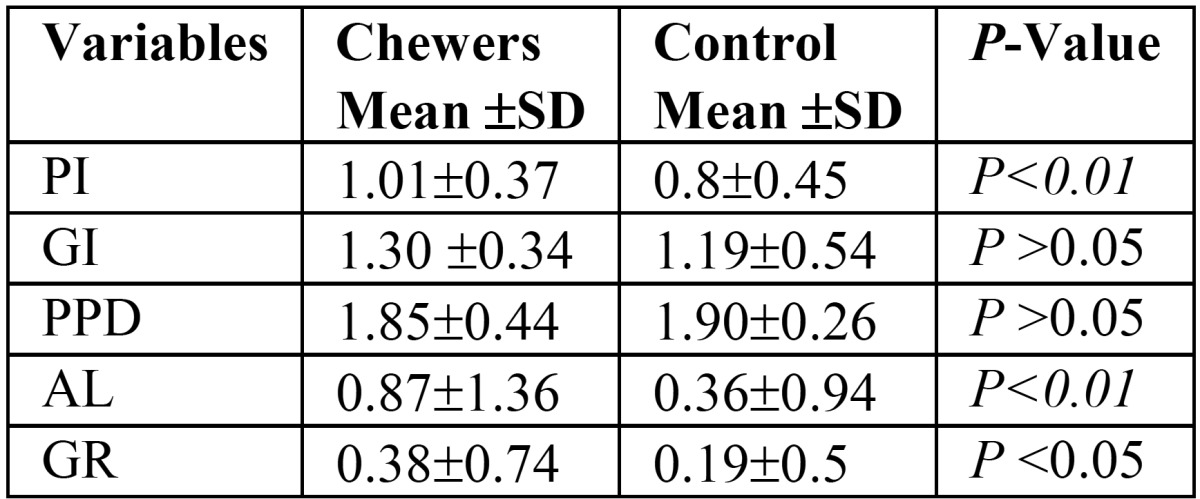
Comparison of periodontal variables in chewers and control.

 Table 4 shows the periodontal parameters in chewing side vs. non-chewing side among khat chewers. Interestingly, the results showed that the chewing side had less dental plaque, less gingivitis, and less probing pockets but more gingival recession than non-chewing side.

**Table 4:**
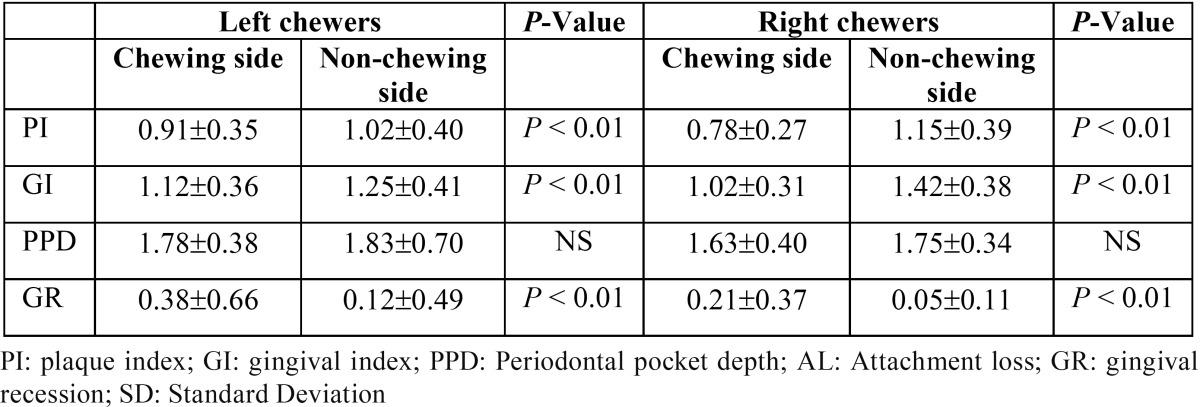
Comparison of periodontal variables in chewing and non-chewing side.

There was a significant positive association between the duration of chewing and most of periodontal parameters (PI, PPD, AL, GR ). Howevere, no significant association was found bewtween the duration of the habit and GI. Likewise, the frequency of chewing showed a significant effect on PPD, AL, and GR, but had no effect on plaque and gingival indices, ( Table 5).

**Table 5:**
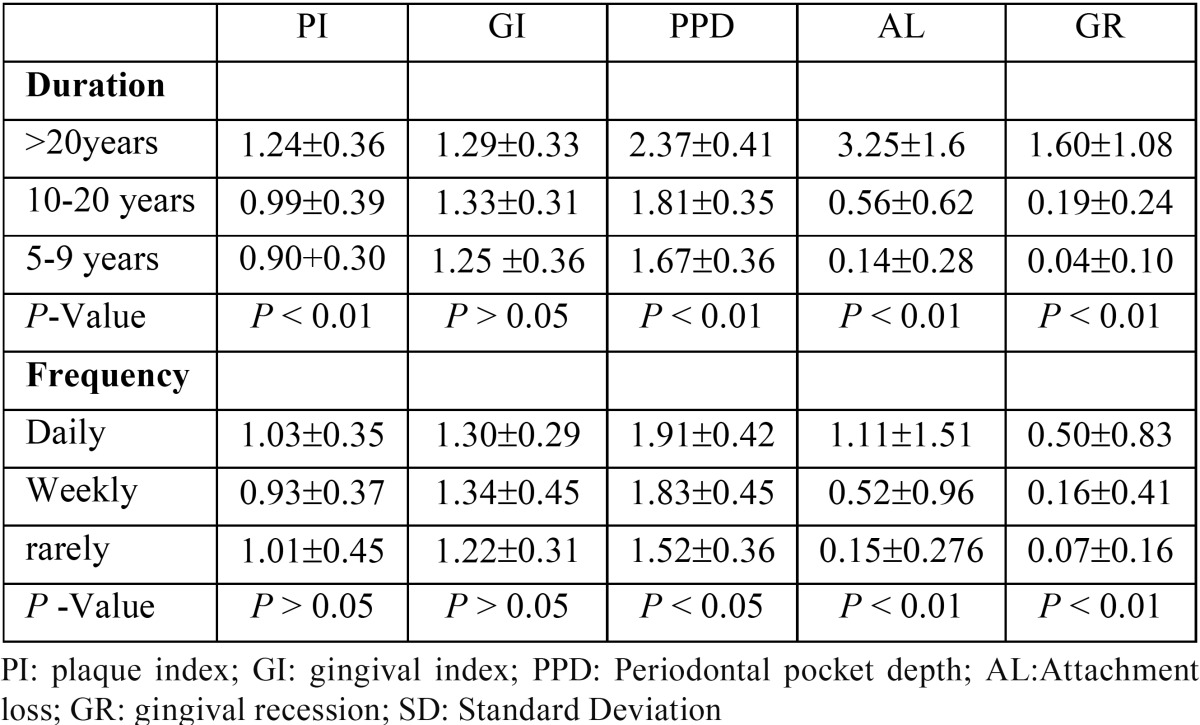
Periodontal variables among khat chewers according to the duration and frequency of chewing.

## Discussion

Khat chewing is widespread habit in Yemen; it is considered as a part of the social fabric of Yemeni society. It is mainly a male predominant habit, although the number of women practicing this habit is steadily on the rise. Studies on the contents and effects of the khat on the physical and mental well-being are rather scarce and fairly recent (1,3,4,8).The present study investigated the effects of chronic khat chewing on various periodontal parameters among male chewers compared to age- and gender- matched control non-chewers. It is worth mentioning here that comparison of our study findings with other studies is difficult due to various factors such as different sampling methods, lack of control in some studies and different examination criteria used.

The results showed poor oral hygiene practices among khat chewers compared with non-chewers. Of total, only 6% of khat chewers reported regularly brushing their teeth compared with around 38% of controls. This was reflected by higher accumulation of plaque among khat chewers than non-chewers. This result is in agreement with another study (13), which reported poorer oral hygiene among khat chewers. However, this result is in contrast to another study conducted in Kenya, which reported less plaque and better oral hygiene among khat chewers than their counterpart controls (16). Interestingly, we found that the chewing sides had significantly less plaque than the opposite sides. This result can be explained by the mechanical cleansing effect of khat on the chewing side together with increased salivary stimulation induced by mastication and sympathomimetic effect of alkaloid content in khat (5,8) .

In the present study, khat chewers and their counterpart controls had comparable gingival indices, with no significant differences between the two groups. Additionally, chewing sides showed less gingival inflammation compared with non-chewing sides, probably due to the mechanical cleansing action exerted by continuous chewing. This finding is consistent with the result of Jorgensen and Kaimenyi (16), which reported less inflamed gingiva among khat chewers than non-chewers. Moreover, a more recent study conducted by Al-Hebshi *et al.*, found khat chewers had lower mean scores gingival index and bleeding on probing than non-chewers, suggesting a beneficial effect of khat on periodontal tissues. The authors suggested that khat constituents may have anti-plaque and anti-gingivitis properties, indicating that khat chewing is probably not detrimental to the periodontal tissues (15). Moreover, there is some evidence suggesting that khat components may have positive influence on oral microbial ecosystem (21,22). A 2005 study on the effect of khat on microbial biofilm reported that khat was associated with higher levels of some health-compatible periodontal bacteria and lower levels of the periodontal pathogens (22). One more recent study showed that subgingival biofilm of Khat chewers with chronic periodontitis harbored less periopathogenic bacteria in both healthy and diseased sites (21). However, these results are inconclusive and more research is still needed to investigate in-depth the influence of khat on oral microflora.

This study revealed that Khat chewers had significantly more attachment loss and more gingival recession than non-chewers, which is in agreement with most of previous studies (5,8,12,13,17). A plausible explanation of these findings is that chronic and intense khat chewing probably results in chronic trauma and vertical impaction to the periodontium, resulting in gingival recession and attachment loss.

Our study demonstrated that khat chewers had slightly lower mean probing pocket depth scores compared to the non-chewers. Such results are expected due to higher gingival recession among chewers than non-chewers, as noted above. Another possible explanation is the fact that the anatomic structures usually maintain better health when subjected to physiologic friction. These findings are in agreement with other studies (5).

Comparing chewing sides with the opposite sides among khat chewers, the chewing side showed more gingival recession but significantly lower mean scores of gingival, plaque and periodontal pocket depth than the opposite sides. These findings should not be interpreted as contradictory findings, but, rather this can be explained in the context that khat chewing mechanically cleanse teeth resulting in less plaque and milder gingival inflammation in the chewing side. On the other hand, the continuous mechanical friction due to khat chewing usually induces gingival recession and attachment loss. Many other investigators also reported similar results (5,8).

Additionally, we observed a positive association between the duration as well as the frequency of chewing with some periodontal variables. These findings are in line with other studies which reported a significant association between the prevalence and severity of periodontal diseases and the duration and intensity of khat chewing (5,10). Ali suggested that the increase in exposure to khat habit will increase the risk of developing periodontal pockets and gum recession (10). The mechanism by which khat chewing deteriorate periodontal health condition is most probably due to heavy occlusal load and trauma resulting from continuous chewing. Moreover, chemical irritation resulting from the chemical substances present in khat is also a possible factor.

Like most of studies this study had several limitations that should be taken into consideration while interpreting the results. The main limitation is the fact that this study did not investigate and control other major risk factors known to be associated with periodontitis, such as smoking, oral hygiene and socioeconomic variables. Therefore, future studies that investigate in depth the effect of khat on periodontal tissues after adjusting all potential confounding factors are highly recommended. Moreover, the generalizability of the results to all khat chewers is questionable, therefore future well-planned large-scaled studies involving both genders are required. Another concerning limitation is the small samples size of the control group, which is attributed to the difficulty in finding non-chewer adult males in a society where over 80% of the people are chewers (5). Additionally, the failure to blind the examiner to khat chewing status might have caused detection bias. Therefore, in future research the examiner can be blinded to the habit history and should examine the oral cavity first to avoid such bias.

## Conclusions

The findings of the present study found that gingival condition and probing pocket depth did not differ significantly between chewers and non-chewers, whearase more plaque accumulation, higher loss of attachment and more gingival recession were associated with khat chewing. Moreovere, the chewing side compared to the opposite side showed less plaque, milder gingivitis and less probing pocket depth but more gingival recession. Additionally, there was a significant association between the duration and the frequency of khat chewing and various periodontal variables.
